# The relationship between work and home characteristics and work engagement in medical residents

**DOI:** 10.1007/s40037-017-0364-y

**Published:** 2017-07-04

**Authors:** Hanne Verweij, Madelon L. M. van Hooff, Frank M. M. A. van der Heijden, Jelle T. Prins, Antoine L. M. Lagro-Janssen, Hiske van Ravesteijn, Anne E. M. Speckens

**Affiliations:** 1 Department of Psychiatry, Radboud University Medical Center, Nijmegen, The Netherlands; 2 Behavioural Science Institute, Radboud University, Nijmegen, The Netherlands; 3 Department of Neuropsychiatry, Vincent van Gogh Institute for Psychiatry, Venlo, The Netherlands; 4 MCL Academy, Medical Center Leeuwarden, Leeuwarden, The Netherlands; 5 Department of Primary and Community Care, Unit Gender and Womens’ Health, Radboud University Medical Center, Nijmegen, The Netherlands

**Keywords:** Work engagement, Medical residents, Demands & resources, Work-home interference

## Abstract

**Introduction:**

Work engagement is important for medical residents and the healthcare organizations they work for. However, relatively little is known about the specific predictors of work engagement in medical residents. Therefore, we examined the associations of work and home characteristics, and work-home interference with work engagement in male and female residents.

**Methods:**

This study was conducted on a nationwide sample of medical residents. In 2005, all Dutch medical residents (*n* = 5245) received a self-report questionnaire. Path analysis was used to examine the associations between the potential predictors and work engagement.

**Results:**

In total, 2115 (41.1%) residents completed the questionnaire. Job characteristics, home characteristics and work-home interference were associated with work engagement. Important positive contributing factors of work engagement were opportunities for job development, mental demands at work, positive work-home interference and positive home-work interference. Important negative contributing factors were emotional demands at work and negative home-work interference. The influence of these factors on work engagement was similar in male and female residents.

**Discussion:**

Opportunities for job development and having challenging work are of high relevance in enhancing work engagement. Furthermore, interventions that teach how to deal skilfully with emotional demands at work and home-work interference are expected to be the most effective interventions to enhance work engagement in medical residents.

## What this paper adds

Work engagement is important for medical residents and the healthcare organizations they work for. However, relatively little is known about the specific predictors in medical residents. Opportunities of job development, mental demands at work and positive work-home interference are important positive contributing factors of work engagement. Emotional demands at work and negative work-home interference are important negative contributing factors of work engagement. In order to enhance work engagement in medical residents it is important to create opportunities for job development and teach residents how to deal with emotional demands at work and home-work interference.

## Introduction

Overall, medical residents are engaged in their work [1]. Work engagement is defined as a positive, fulfilling, work-related state of mind that is characterized by vigour (high levels of energy while working), dedication (strongly involved in work), and absorption (fully concentrated and happily engrossed in work) [2]. Work engagement is related to being more committed to the organization, having less turnover intentions, more satisfied clients and better financial outcomes [3, 4]. Within the medical sector work engagement is related to making fewer medical errors. Highly engaged residents reported fewer errors due to inexperience and/or lack of time than those who were less engaged [5]. Although job demands and work pressure are high in hospitals, and stress and burnout are common in medical residents, one-fourth of the residents can still be characterized as highly engaged [1]. Why are some residents more engaged in their work than others? How can we increase work engagement in medical residents? In order to stimulate work engagement in medical residents, we should gain a better understanding of potential contributors of work engagement in this specific population.

According to the Job Demands and Resources theory and previous studies, job resources are positively related to work engagement [6, 7]. Job resources refer to those aspects of the job that are functional in achieving work goals, reduce job demands and the associated physiological and psychological costs, and/or stimulate personal growth and development [8]. Important resources are opportunities for development, performance feedback, autonomy, skill variety, and social support from colleagues and supervisors. However, the specific resources that predict work engagement may differ per organization [9]. In addition to job resources, job demands also seem to be related to work engagement [10]. Job demands refer to work characteristics that require physical and/or psychological effort and are associated with certain physiological or psychological costs [8]. Although demands are associated with costs and often related to negative work outcomes, job demands that are challenging such as job responsibility, workload, and time urgency might also be positively related to work engagement [11]. However, a study by Mache et al. [10] in 123 surgeons found that job demands were negatively related to work engagement, and that job resources had a greater impact on surgeons’ work engagement than job demands. Especially having influence at work, opportunities for development and social support seemed to contribute to work engagement in surgeons. Furthermore, several studies indicate that interaction between demands and resources is relevant as well, suggesting that job resources become more salient and gain their motivational potential when employees are confronted with high job demands [6, 12].

Less is known about the potential influence of home characteristics on work engagement, but several studies indicated that one’s functioning at home might impact one’s functioning at work and vice versa [13–16]. A study by Bakker et al. [17] found that home resources and demands were associated with work engagement in a sample of 323 Dutch dual-earner couples, whereas a longitudinal study by Hakanen et al. [18] did not find an effect of home demands or resources on work engagement in Finnish dentists (*n* = 2555).

The interference between the work and home domain might also be related to work engagement [19, 20]. This work-home interference and home-work interference can be experienced negatively when demands from the work and family roles are incompatible, such that participation in one role makes it difficult to participate in the other, and positive when positive experiences from one role make it easier to enhance participation in the other role [21, 22]. A study by Montgomery et al. [19] found that positive interference between the home and work domain was correlated with feelings of dedication at work.

With the increasing percentage of women within the medical profession it is very relevant to examine gender differences in work engagement and their contributing factors [23]. Prins et al. [1] indicated that female residents report significantly less vigour than male residents. Furthermore, Verweij et al. [24] found gender differences in contributing factors to burnout. In females, social support from family or partner seemed protective against burnout. In males, social support from colleagues and participation in decision-making at work seemed more important. As burnout and work engagement are negatively correlated, we might suspect that there are gender differences in work engagement as well. In addition, Rothbard [25] found that a positive affect towards the home/family life was related to work engagement only among women. This could indicate that the potential contributors of work engagement might differ between male and female residents.

The aim of the present study was to test a model including all the potential contributing factors of work engagement as described before, in order to examine which factors are most important. This will inform us about how to enhance work engagement. In addition, we explored whether there are gender differences with regard to possible contributing or protective factors of work engagement. We formulated the following hypotheses:

### Hypothesis 1

We expect (a) the job and home demands to be negatively related to work engagement, (b) the job and home resources to be positively related to work engagement, (c) the job/home resources to be more strongly related to work engagement if job/home demands are high, (d) positive work-home/home-work interference to be positively related to work engagement, and negative work-home/home-work interference to be negatively related to work engagement.

### Hypothesis 2

We expect the model to be different for male and female residents; we expect the home demands and resources to be more strongly related to work engagement in female residents than in male residents.

## Methods

### Participants and procedure

This study was conducted in a large sample of Dutch medical residents. Data were collected in a previous study aimed at gaining insight into the prevalence of burnout and work engagement in medical residents in the Netherlands [1]. All 5245 Dutch medical residents in training on 1 October 2005 were invited to take part in the survey. They received a self-report questionnaire at their home address and they could choose to complete the questionnaire anonymously by hand or online. Participation was voluntary. A cover letter was attached and explained the purpose of the study and emphasized anonymity. All residents were sent three reminders and a non-response form.

At the time of data collection, ethical approval was not required. However, all participants were informed about the study, participation was voluntary and anonymity was guaranteed, also by using a third party to blind the respondents. We ensured that no possible harm could come to the participants of our study.

### Measures

#### Demographics and occupational characteristics

Respondents provided information on: gender; age; type of specialty and number of years in training.

#### Work engagement

We used the Utrecht Work Engagement Scale to measure work engagement [26]. This questionnaire consists of 15 items measuring three engagement subscales: *Vigour *(5 items; α = 0.80), *dedication* (5 items; α = 0.88), and *absorption* (5 items; α = 0.78). Items were rated on a 7-point Likert scale ranging from 0 ‘never’ to 6 ‘always’. Example items: ‘At my job, I feel bursting with energy.’ (vigour); ‘My job inspires me.’ (dedication); ‘When I am working, I forget everything else around me.’ (absorption).

#### Job demands

*Workload* (4 items, α = 0.87), *emotional demands at work* (6 items, α = 0.79) and *mental demands at work* (4 items, α = 0.77) were each measured by scales of the Questionnaire on the Experience and Evaluation of Work [27]. Items were rated on a 5-point Likert scale ranging from 1 ‘never’ to 5 ‘always’. Example items: ‘Do you have to work very fast?’ (workload); ‘Is your work emotionally demanding?’ (emotional demands), and ‘Does your work demand a lot of concentration?’ (cognitive demands).

#### Job resources

Six job resources were measured using scales of the Questionnaire on the Experience and Evaluation of Work [27]: *Job autonomy* (3 items, α = 0.73), *job development* (3 items, α = 0.80), *social support from colleagues* (3 items, α = 0.84), *performance feedback* (5 items, α = 0.83), *supervisory coaching* (6 items, α = 0.86), and *participation in decision making* (4 items, α = 0.77). All items were scored on a 5-point rating scale ranging from 1 (‘never/poor/totally disagree’) to 5 (‘always/good/totally agree’). Example items: ‘Do you have freedom in carrying out your work activities?’ (autonomy); ‘At work I am given the opportunity to develop my personal strengths’ (job development); ‘Can you, when necessary, ask your colleagues for help?’ (social support from colleagues); ‘I receive enough feedback from my supervisor in regards to my work’ (performance feedback); ‘My supervisor uses his/her influence to help me solve my problems at work’ (supervisory coaching); and ‘I feel that I am involved in making important decisions’ (participation in decision making).

#### Home demands

Three home demands were measured using scales used in previous studies [19, 28]: *Homeload* (5 items, α = 0.75), *emotional demands *(3 items, α = 0.76) and *mental demands *(3 items, α = 0.88). All items were scored on a 5-point rating scale ranging from 1 (‘never’) to 5 (‘always’). Example items: ‘Do you have to carry out a lot of tasks at home [household/caring tasks]?’ (homeload); ‘Are you confronted with situations in your private life that are emotionally charged?’ (emotional demands); and ‘Do you have to plan and organize a lot of things in relation to your home life?’ (mental demands).

#### Home resources

Three home resources were measured using scales used in previous studies [15, 17]: *personal autonomy* (4 items, α = 0.82), *social support from partner/family *(4 items, α = 0.87), and *opportunity for personal development* (3 items, α = 0.88). All items were scored on a 5-point rating scale ranging from 1 (‘never’) to 5 (‘always’). Example items: ‘I manage daily life at home’ (personal autonomy); ‘My family/partner pays attention to my feelings and problems’ (social support from partner/family); and ‘I can develop my talents during my free time’ (opportunity for personal development).

#### Work-home interference

The Survey Work-home Interaction NijmeGen (SWING) was used to measure work-home interference [29]. This questionnaire measures four subscales: *positive work-home interference* (3 items, α = 0.42), *negative work-home interference* (3 items, α = 0.73), *positive home-work interference* (3 items, α = 0.68), and *negative home-work interference* (3 items, α = 0.79). All items were scored on a 5-point rating scale ranging from 1 (‘never’) to 5 (‘always’). Example items: ‘How often does it happen that after a pleasant working day, you feel more in the mood to engage in activities with your spouse/family/friends?’ (positive work-home interference); ‘How often do you find it difficult to fulfil your domestic obligations because you are constantly thinking about your work?’ (negative work-home interference); ‘How often does it happen that after spending a pleasant weekend with your spouse/family/friends, you have more fun in your job?’ (positive home-work interference); and ‘How often do you not fully enjoy your work because you worry about your home situation?’ (negative home-work interference).

## Data analysis

We analyzed the data by means of path analysis, using the Mplus7 statistical software package [30]. Path analysis is a subset of structural equation modelling using only measured variables and no latent variables. We developed a model and examined the associations between the specific work and home demands and resources and the four types of work-home interference on the one hand, and the three work engagement subscales on the other hand. These associations or pathways in the models represent hypotheses, which are based on previous research and theoretical propositions as described in the introduction. We included work characteristics in our analyses first, and added home characteristics in a next step, in order to be able to examine if including home characteristics explained an additional proportion of the variance in work engagement, beyond the effects of work characteristics. To examine possible gender differences in these associations we employed multi-group path analysis in Mplus7 [30]. The improvement in fit of the models was assessed using the chi-square difference test, the root mean square error of approximation (RMSEA) and the comparative fit index (CFI). Values of 0.90 and higher (CFI) and 0.08 or lower (RMSEA) indicate an acceptable fit [31]. Because of the large dataset and the number of variables included in this model we chose to indicate *p*‑values below 0.01 as significant in order to decrease the chance of type 1 errors. Standardized path coefficients (Beta values) were calculated to determine the possible predicting factors of work engagement. These indicate the patterns of associations between the predicting factors and work engagement. Standardized path coefficients (β) with values of less than 0.10 can be interpreted as small effects, values of around 0.30 can be interpreted as medium effects and values above 0.50 can be interpreted as large effects [32].

Before testing our hypotheses, the items representing the constructs in the research model were subjected to confirmatory factor analyses using Mplus7. All measures we used were employed in previous studies and generally proved to be valid. The fit indices of the measurement model were near adequate, χ^2^(3444) = 17,382.40, RMSEA = 0.04, CFI = 0.85, standardized root mean residual = 0.05. All items loaded significantly on the predicted factor. However, one item from the homeload construct had a low factor loading (<0.40). We decided not to remove this item because of its conceptual importance and because the internal consistency of the construct, expressed with Cronbach’s α coefficient, was considered sufficient (α = 0.75).

## Results

### Study population

Of the 5245 residents who were invited, 105 indicated that they were no longer residents. Of the remaining 5140 residents, 125 (2.4%) indicated that they did not wish to participate. In total, 2115 (41.1%) completed the questionnaire [1]. Characteristics of the respondents are presented in Table 1.

**Table 1:** Characteristics of the respondents (*n* = 2115)

Variable	Mean (SD)	*N*	%
*Gender*			
Female		1290	61.0
Male		820	38.8
Missing		5	0.2
*Age, range 23–58 years*	31.5 (3.5)		
*Years in training*	3.0 (1.5)		
*Medical specialty in groups*		2115	
(Internal) medical specialties		951	45.0
Surgical specialties		649	30.7
Supportive/diagnostic specialties		270	12.8
Psychiatry		242	11.4
Missing		3	0.1

*SD* standard deviation

### General model for work engagement

Table 2 provides an overview of the fit indices for the models that were estimated. In the first model (M1), only job demands and job resources were modelled to be related to engagement. As indicated by the CFI and RMSEA value, this model fitted well with the data. Building on this first model, we estimated a second model (M2) that was identical to M1, except that it additionally included the associations between home demands and home resources and engagement. As can be seen in Table 2, adding these paths significantly improved the fit of our model. In Model 3 (M3), we extended M2 by including the interactions between job demands and job resources, in order to examine the proposition of the Job Demands and Resources model that demands moderate the association between resources and engagement [33]. In order to avoid problems of multicollinearity, these interactions were computed using mean-centred scores [34]. As M3 did not provide a better fit than M2, these interactions were omitted from further analyses. Model 4 (M4) was therefore also based on M2, and included the interactions between home demand and home resources. Again, this model did not improve model fit compared with M2. Therefore, our final model (M5), in which the paths between positive and negative home-work and work-home interference and engagement were estimated, was also based on M2. This model not only fitted significantly better than M2, but also showed a very good fit in an absolute sense.

**Table 2:** Fit indices for different models

	χ2 (df)	∆ χ2 (df)	RMSEA	CFI
M1: work demands and work resources as predictors of work engagement	438.85 (111)		0.04	0.93
M2: M1 + home demands and home resources as predictors of work engagement	360.22 (93)	78.63 (18)^a^	0.04	0.94
M3: M2 + interactions work demands × work resources as predictors of work engagement	317.11 (39)	43.11 (54)	0.06	0.94
M4: M2 + interactions home demands × home resources as predictors of work engagement	326.44 (6)	33.78 (27)	0.05	0.94
M5: M2 + WHIpos, WHIneg, HWIpos, and HWIneg as predictors of work engagement	82.39 (81)	277.83 (12)^a^	0.00	1.00
M6: M5 + Gender. Equality constraints on all structural paths	249.91 (219)		0.01	0.99
M7: M5 + Gender. No constraints for paths	200.65 (162)	49.26 (57)	0.02	0.99

*WHI* work-home interference, *HWI* home-work interference, *RMSEA* root mean square error of approximation, *CFI* comparative fit index

^a^Statistically significant at *p* < 0.01

### Gender differences in the work engagement model

Results of the gender-specific analyses are presented in Table 2. We started by modelling the paths that were included in M5 in female and male residents separately. Initially, we imposed equality constraints on all structural paths, thus assuming the strength of all associations to be similar for both sexes. This model provided a good fit to the data (M6). Subsequently, in a second model (M7) we removed these equality constraints to examine if this resulted in a better fitting model. Table 2 shows that M7 (without constraints) did not fit better than M6 (with constraints). Therefore, our data do not provide evidence for gender differences in the associations between work and home characteristics and work-home interference, and engagement.

### Contributing and protective factors of engagement

**Fig. 1:**
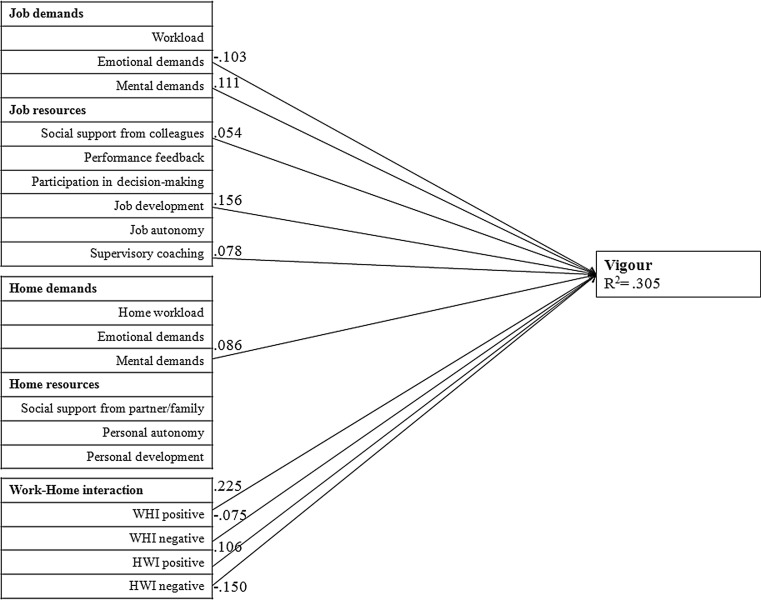
Path analysis of vigour. Only associations that were statistically significant at *p* < 0.01 are presented. (*WHI* work-home interference, *HWI* home-work interference)

**Fig. 2:**
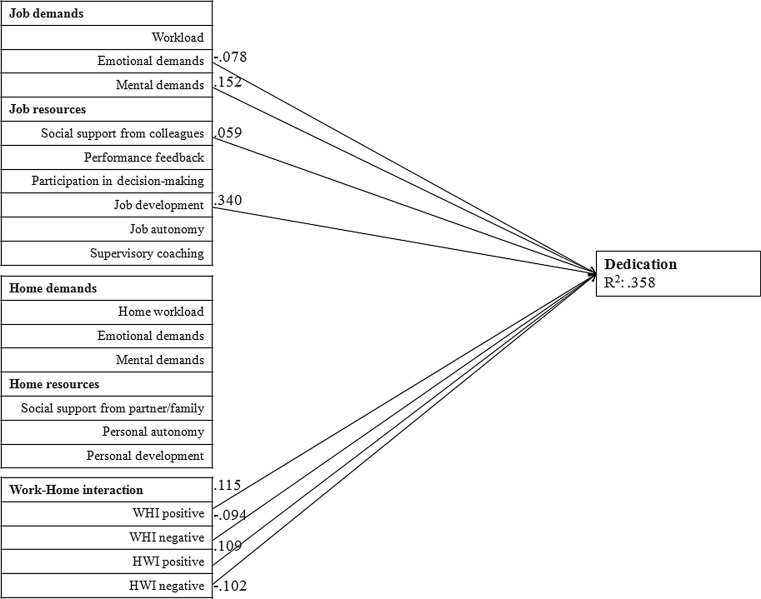
Path analysis of dedication. Only associations that were statistically significant at *p* < 0.01 are presented. (*WHI* work-home interference, *HWI* home-work interference)

**Fig. 3:**
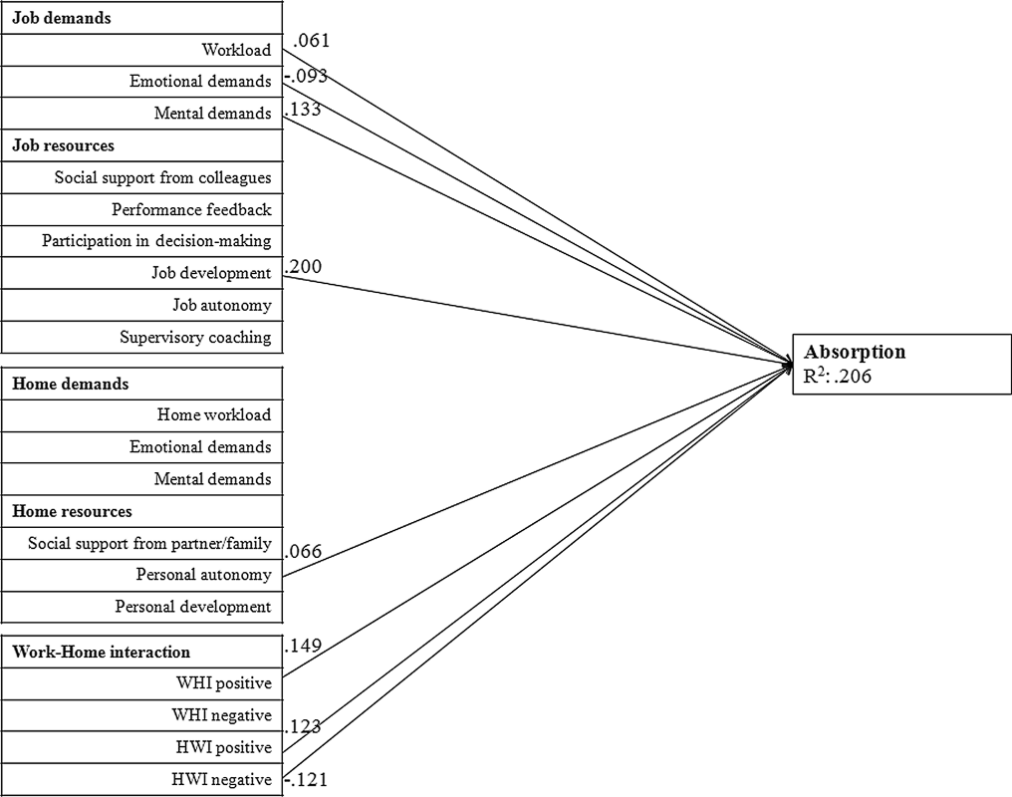
Path analysis of absorption. Only associations that were statistically significant at *p* < 0.01 are presented. (*WHI* work-home interference, *HWI* home-work interference)

Fig. 1, 2 and 3 provide the graphical representation of the final model (M5). In order to reduce the complexity of the figure, we divided the figure into three separate figures, each representing one subscale of work engagement. Furthermore, the figures only show the significant associations and standardized path coefficients (Beta values). The factors in this model explain a significant proportion of variance of vigour (R^2^ = 0.305), dedication (R^2^ = 0.358) and absorption (R^2^ = 0.206). All significant associations of the three subscales of work engagement are described below.

#### Vigour

With regard to the positive associations with vigour, we found that mental demands at work, social support from colleagues, job development, supervisory coaching, mental demands at home, positive work-home interference and positive home-work interference were positively associated with vigour. Emotional demands at work, negative work-home interference and negative home-work interference were negatively associated with vigour.

#### Dedication

Mental demands at work, job development, social support from colleagues, positive work-home interference and positive home-work interference were positively associated with dedication. Emotional demands at work, negative work-home interference and negative home-work interference were negatively associated with dedication.

#### Absorption

Mental demands at work, job workload, job development, personal autonomy, positive work-home interference and positive home-work interference were positively related to absorption. Emotional demands at work and negative home-work interference were negatively associated with absorption.

## Discussion

The aim of this study was to examine the associations of work and home characteristics and work-home interference with work engagement in Dutch male and female medical residents.

The results partly confirmed our hypothesized model in that we found work demands and resources, home demands and resources, and work-home and home-work interference to be associated with work engagement. However, we did not find support for hypothesis 1c; demands did not moderate the association between resources and engagement. Furthermore, our data do not provide any evidence for possible gender differences in the associations between work and home characteristics and work-home interference, and engagement (hypothesis 2).

Examining these associations in more detail, we see that especially opportunities for job development, positive work-home interference, positive home-work interference and mental demands at work were important contributing factors to all three subscales of work engagement. Interestingly, ‘mental demands at work’ was positively rather than negatively related to work engagement, suggesting that mental demands are considered to be challenging rather than exhausting [11]. Emotional demands at work and negative home-work interference had an important negative impact on work engagement.

There was only a very small effect of home demands and resources on work engagement. The work-home interferences, both the negative and positive home to work interference and work to home interference, seemed more important for experiencing work engagement. This might indicate that for medical residents the home situation in itself is not that influential, but the interference between the home and work domain has a greater impact on engagement at work.

Furthermore, we did not find gender differences regarding the associations in the model. This is in contrast with our expectations, but it is in line with research in a heterogeneous occupational sample (*n* = 846) by Korunka et al. [35], who did not find gender differences either in the relationship between job resources and work engagement. The strengths of the associations between resources, demands and work-home interferences with work engagement in our research are similar for male and female residents. However, previous research in medical residents demonstrated that female residents experienced more home demands and more negative home-to-work interference, while male residents reported more social support from colleagues and supervisory coaching [24]. The circumstances seemed slightly better for male residents as they reported more of what is needed for being engaged, which might explain that male residents also reported more vigour compared with female residents [1].

Although, more insight into the specific contributing factors of work engagement might lead to a better functioning of the residents themselves, the organization and a higher quality of patient care, it is also important to be aware of the possible downsides of work engagement [36]. Bakker et al. [37] suggested that there might be a limit to engagement. Overly engaged workers may often work overtime, forget to rest or maintain their personal relationships and hence experience more work-family conflict. In a medical culture with its emphasis on commitment, the high responsibility in patient care and expectation to work overtime, this is important to bear in mind.

### Strengths and limitations

One of the strengths of this study is the large and representative population. All medical residents in the Netherlands were invited to participate, which resulted in a large sample with residents from different regions of the country, different hospital settings and different medical specialties.

As any study, the present study also has limitations. First of all, the data were collected at the end of 2005. So, our findings might have been overhauled by social or cultural changes that have since taken place or by developments in the postgraduate training of medical residents. However, we believe that although in 10 years the absolute values of work engagement and its contributors might have slightly changed, the relationships between these variables and work engagement have not been affected by such changes. The Job Demands-Resources model, on which we based our hypotheses, assumes that the availability of job resources helps in dealing with job demands and plays a motivational role, which fosters work engagement [38]. There is no reason to believe that this process or the specific characteristics associated with work engagement in medical residents have changed during the past decade because the nature of the job has not essentially changed.

Secondly, the data were collected at one point in time, so the associations cannot be interpreted causally. For instance, it could also be that medical residents with high levels of engagement more easily identify positive work-home interference. However, longitudinal research by Hakanen et al. [18] did not find evidence for the reversed effect of work engagement on job demands and resources.

Thirdly, although considering the nature of the survey the response rate was quite high (41%), more than half of the invited medical residents did not respond. This could have led to a selection bias. Common reasons for not responding in those who took the trouble to send in their non-response form appeared to be a lack of time (22%) and a lack of energy (11%). This might have resulted in an overestimation of work engagement in medical residents. However, again, this might especially have affected the absolute levels of burnout and its contributors instead of their relationships. Furthermore, as all measures were based on self-reports, common method variance may have biased our findings. Ideally, future research should combine self-report questionnaires with more objective measures, such as computerize tasks, observations and organisation’s records.

### Future research and practical implications

Future research should use longitudinal designs to better explore the predictive nature of the associations found in our model and the possible long-term consequences of work engagement in relationship with health. Furthermore, forthcoming studies about work engagement would benefit from a focus on interventions, and study the impact and effectiveness of these interventions.

With regard to implications for clinical practice, our findings support the improvement of resources at work, such as better opportunities for career development, personal development and creating a supportive culture. Furthermore, residents should learn how to deal with emotional demands at work, for example by facilitating regular supervision or peer supervision where junior doctors can exchange difficult situations they might have encountered. Alternatively, one might stimulate curricular initiatives that incorporate aspects of allowing and regulating emotions, such as narrative medicine or mindfulness training [39, 40]. In addition to changing circumstances at work, there should be more attention to the benefits of the decreasing negative interference and stimulating positive interference between home and work. Hospitals could provide facilities supporting the work-life balance such as flexible working hours, self-scheduling, part-time work, and childcare. Secondly, and this might even be more important, hospitals should create a culture in which residents feel supported in using these facilities without experiencing a negative effect on their career opportunities [41].
